# The genome sequence of the Eurasian common lizard, *Zootoca vivipara* (von Jacquin, 1787) (Squamata: Lacertidae)

**DOI:** 10.12688/wellcomeopenres.26692.1

**Published:** 2026-06-11

**Authors:** Kathryn R Elmer, John L Smout, Wellcome Sanger Institute Tree of Life Management, Samples and Laboratory team, Wellcome Sanger Institute Scientific Operations: Sequencing Operations, Wellcome Sanger Institute Tree of Life Core Informatics team, Tree of Life Core Informatics collective, Darwin Tree of Life Consortium

**Affiliations:** 1 University of Glasgow College of Medical Veterinary and Life Sciences, Glasgow, Scotland, UK

**Keywords:** Zootoca vivipara, Eurasian common lizard, genome sequence, chromosomal, Squamata

## Abstract

We present a genome assembly from an individual male *Zootoca vivipara* (Eurasian common lizard; Chordata; Lepidosauria; Squamata; Lacertidae). The assembly contains two haplotypes with total lengths of 1 424.51 megabases and 1 423.70 megabases. Most of the haplotype 1 assembly (99.49%) and the haplotype 2 assembly (99.42%) are each scaffolded into 18 chromosomal pseudomolecules, including the Z _1_ and Z _2_ sex chromosomes. The mitochondrial genome has also been assembled, with a length of 17.09 kilobases. This assembly was generated as part of the Darwin Tree of Life project, which produces genomes for eukaryotic species found in Britain and Ireland.

## Species taxonomy

Eukaryota; Metazoa; Chordata; Lepidosauria; Squamata; Lacertidae; *Zootoca*; *Zootoca vivipara* (von Jacquin, 1787) (NCBI:txid8524).

## Background

The Eurasian common lizard, *Zootoca vivipara*, is a small bodied lacertid and one of the few lizard species found natively in Great Britain and Ireland. Common lizards tend to occupy boggy heathland, meadows, and woodland edges. In Britain it is has a patchy distribution, being rare in many regions and habitats despite having overall an extensive geographic distribution. In fact, *Z. vivipara* has the broadest expanse and most northerly reach of any terrestrial reptile. The species spans Eurasia, from Ireland in the west to Japan in the east, to the Alps and Pyrenees in the south of Europe and as far north as the Arctic Circle. There are six phylogeographic lineages of *Z. vivipara* across this Eurasian distribution ( Recknagel *et al.*, 2018). The Western Viviparous lineage, which is distributed throughout Western Europe, is the lineage found in Great Britain and Ireland. The species is listed as a least concern species by IUCN because it is widespread, though there are local impacts on populations due to habitat loss ( Beebee & Ratcliffe, 2018).

A striking feature of the species is that among the six phylogeographic lineages there are two parity modes – oviparous, which is the ancestral state, and viviparous reproduction. This represents one of the most recent evolutions of live birth that is known in any amniote ( Recknagel *et al.*, 2021b). Across almost the entire distribution (including in Britain and Ireland) the common lizard is viviparous. The oviparous lineages are found in the mountainous southern portion of the range, in the Pyrenees and the Alps ( Horreo *et al.*, 2018). Some researchers propose that there are multiple subspecies in *Zootoca vivipara* based on karyotype variation and the different parity modes ( Cornetti *et al.*, 2014; Kupriyanova & Melashchenko, 2011). The oviparous and the viviparous lineages are closely related and are morphologically similar despite differing in parity mode ( Recknagel *et al.*, 2018; Roitberg *et al.*, 2025). Hybridisation can occur when viviparous and oviparous lineages come into contact ( Recknagel *et al.*, 2018) or are crossed in experimental conditions ( Arrayago *et al.*, 1996), but throughout most of their range they do not overlap and hybridisation is extremely rare ( Cornetti *et al.*, 2015). This bimodal aspect of their reproduction makes the common lizard a very important model for studying the evolution of parity modes and life history strategies.

The species lives a highly seasonal existence, which is especially restricted when inhabiting northern latitudes or high altitudes. They hibernate underground throughout the winter, though they can occasionally emerge to bask during mild weather during that period ( Hodges & Seabrook, 2022). Mating occurs upon emergence from hibernation in the spring; later in summer, the females lay several offspring in a clutch consisting of calcified eggs in the case of the oviparous females or uncalcified egg sacs from viviparous females (lecithotrophic viviparity) ( Recknagel *et al.*, 2021a). Neonates are small and almost completely black in coloration when they are born. After their first hibernation, most populations spend the next summer as non-reproductive juveniles and the begin sexually reproducing the following year ( McInerny & Minting, 2016). Subadults are overall dark brown, usually lacking dorsal patterning, tending towards black at the tail ( McInerny & Minting, 2016). In adults, the typical colouration is brown on the dorsal area with some patterning of darker and paler stripes or broken dashes. The ventral colouration varies from creamy white through to yellow or orange depending on the sex and location, and this variation has been associated with alternative behavioural strategies ( Vercken *et al.*, 2007).

Common lizards are one of the most intensively studied lizard species in Europe and there is a large scientific community who will benefit from these genome resources. A complete genome sequence for the species will be extremely valuable for reconstructing evolutionary relationships and identifying the genetic basis of variable traits, such as those associated with reproduction and colouration, the structure and evolution of its karyotype and sex chromosomes across the lineages, dispersal and metapopulation analyses, and adaptation to environmental changes.

## Methods

### Sample acquisition

**Figure 1.**
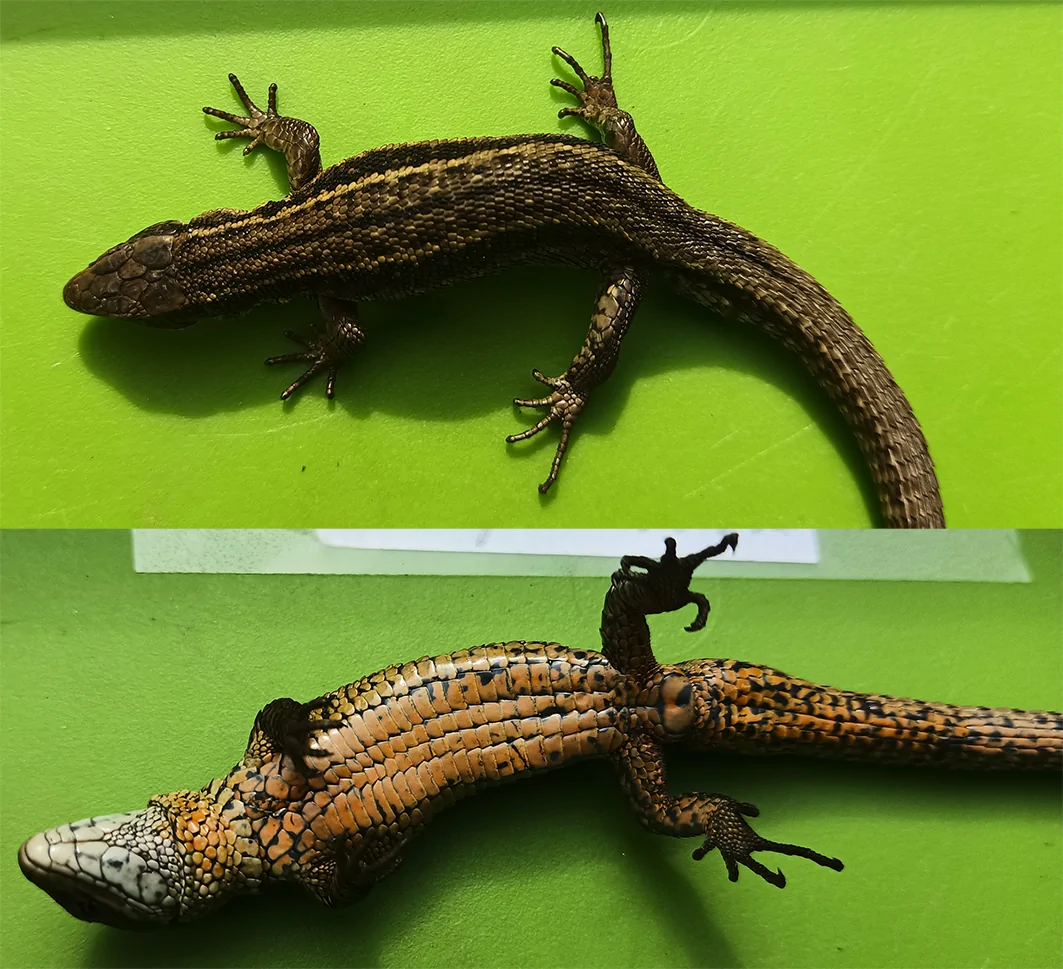
Dorsal and ventral photographs of the male *Zootoca vivipara* (rZooViv2) specimen.

The specimen used for genome sequencing was an adult male *Zootoca vivipara* (specimen ID SAN00002413, ToLID rZooViv2; Figure 1), collected from the Isle of Cumbrae, Scotland, United Kingdom (latitude 55.7759, longitude −4.9323) on 2021-06-14. A female specimen collected on the same occasion was used for RNA sequencing (specimen ID SAN00002412, ToLID rZooViv1), and was also used to produce a second genome sequence (GCA_963506605.1). Lizards were collected from stony walls and verge alongside the road where it meets fields. The specimens and sexes were identified by gross morphology, colouration, and geographic location. For tissue collection, the lizards were euthanised in accordance with Home Office schedule 1 method of stunning by concussion followed by destruction of the brain before tissue sampling took place. Samples were taken from the animals by dissection and preserved in Eppendorf tubes in liquid nitrogen.

### Nucleic acid extraction

Detailed protocols for nucleic acid extraction developed at the Wellcome Sanger Institute (WSI) Tree of Life Core Laboratory are available on protocols.io ( Howard *et al.*, 2025). The specimen used for genome sequencing (specimen ID SAN00002413, ToLID rZooViv2) was weighed and triaged to determine the appropriate extraction protocol. Tissue from the muscle was homogenised by powermashing using a PowerMasher II tissue disruptor. High molecular weight (HMW) DNA was extracted using the Automated MagAttract v3 protocol. DNA was sheared into an average fragment size of 12–20 kb following the Megaruptor®3 for LI PacBio protocol. Sheared DNA was purified by automated SPRI (solid-phase reversible immobilisation). The concentration of the sheared and purified DNA was assessed using a Nanodrop spectrophotometer and Qubit Fluorometer using the Qubit dsDNA High Sensitivity Assay kit. Fragment size distribution was evaluated by running the sample on the FemtoPulse system. For this sample, the final post-shearing DNA had a Qubit concentration of 12.5 ng/μL and a yield of 1 625.00 ng.

RNA was extracted from muscle tissue of the RNA specimen (ToLID rZooViv1) in the Tree of Life Laboratory at the WSI using the RNA Extraction: Automated MagMax™ *mir*Vana protocol. The RNA concentration was assessed using a Nanodrop spectrophotometer and a Qubit Fluorometer using the Qubit RNA Broad-Range Assay kit. Analysis of the integrity of the RNA was done using the Agilent RNA 6000 Pico Kit and Eukaryotic Total RNA assay.

### PacBio HiFi library preparation and sequencing

Library preparation and sequencing were performed at the WSI Scientific Operations core. Libraries were prepared using the SMRTbell Prep Kit 3.0 (Pacific Biosciences, California, USA), following the manufacturer’s instructions. The kit includes reagents for end repair/A-tailing, adapter ligation, post-ligation SMRTbell bead clean-up, and nuclease treatment. Size selection and clean-up were performed using diluted AMPure PB beads (Pacific Biosciences). DNA concentration was quantified using a Qubit Fluorometer v4.0 (ThermoFisher Scientific) and the Qubit 1X dsDNA HS assay kit. Final library fragment size was assessed with the Agilent Femto Pulse Automated Pulsed Field CE Instrument (Agilent Technologies) using the gDNA 55 kb BAC analysis kit.

The sample was sequenced on a Revio instrument (Pacific Biosciences). The prepared library was normalised to 2 nM, and 15 μL was used for making complexes. Primers were annealed and polymerases bound to generate circularised complexes, following the manufacturer’s instructions. Complexes were purified using 1.2X SMRTbell beads, then diluted to the Revio loading concentration (200–300 pM) and spiked with a Revio sequencing internal control. The sample was sequenced on a Revio 25 M SMRT cell. The SMRT Link software (Pacific Biosciences), a web-based workflow manager, was used to configure and monitor the run and to carry out primary and secondary data analysis.

### Hi-C

***Sample preparation and crosslinking***

The Hi-C sample was prepared from 20–50 mg of frozen muscle tissue from the rZooViv2 sample using the Arima-HiC v2 kit (Arima Genomics). Following the manufacturer’s instructions, tissue was fixed and DNA crosslinked using TC buffer to a final formaldehyde concentration of 2%. The tissue was homogenised using the Diagnocine Power Masher-II. Crosslinked DNA was digested with a restriction enzyme master mix, biotinylated, and ligated. Clean-up was performed with SPRISelect beads before library preparation. DNA concentration was measured with the Qubit Fluorometer (Thermo Fisher Scientific) and Qubit HS Assay Kit. The biotinylation percentage was estimated using the Arima-HiC v2 QC beads.

***Hi-C library preparation and sequencing***

Biotinylated DNA constructs were fragmented using a Covaris E220 sonicator and size selected to 400–600 bp using SPRISelect beads. DNA was enriched with Arima-HiC v2 kit Enrichment beads. End repair, A-tailing, and adapter ligation were carried out with the NEBNext Ultra II DNA Library Prep Kit (New England Biolabs), following a modified protocol where library preparation occurs while DNA remains bound to the Enrichment beads. Library amplification was performed using KAPA HiFi HotStart mix and a custom Unique Dual Index (UDI) barcode set (Integrated DNA Technologies). Depending on sample concentration and biotinylation percentage determined at the crosslinking stage, libraries were amplified with 10–16 PCR cycles. Post-PCR clean-up was performed with SPRISelect beads. Libraries were quantified using the AccuClear Ultra High Sensitivity dsDNA Standards Assay Kit (Biotium) and a FLUOstar Omega plate reader (BMG Labtech).

Prior to sequencing, libraries were normalised to 10 ng/μL. Normalised libraries were quantified again to create equimolar and/or weighted 2.8 nM pools. Pool concentrations were checked using the Agilent 4200 TapeStation (Agilent) with High Sensitivity D500 reagents before sequencing. Sequencing was performed using paired-end 150 bp reads on the Illumina NovaSeq X.

### RNA library preparation and sequencing

Libraries were prepared using the NEBNext ^®^ Ultra™ II Directional RNA Library Prep Kit for Illumina (New England Biolabs), following the manufacturer’s instructions. Poly(A) mRNA in the total RNA solution was isolated using oligo (dT) beads, converted to cDNA, and uniquely indexed; 14 PCR cycles were performed. Libraries were size-selected to produce fragments between 100–300 bp. Libraries were quantified, normalised, pooled to a final concentration of 2.8 nM, and diluted to 150 pM for loading. Sequencing was carried out on the NextSeq 500, generating paired-end reads.

### Genome assembly

Prior to assembly of the PacBio HiFi reads, a database of *k*-mer counts ( *k* = 31) was generated from the filtered reads using FastK. GenomeScope2 ( Ranallo-Benavidez *et al.*, 2020) was used to analyse the *k*-mer frequency distributions, providing estimates of genome size, heterozygosity, and repeat content.

The HiFi reads were assembled using Hifiasm in Hi-C phasing mode ( Cheng *et al.*, 2021, 2022), producing two haplotypes. Hi-C reads ( Rao *et al.*, 2014) were mapped to the primary contigs using bwa-mem2 ( Vasimuddin *et al.*, 2019). Contigs were further scaffolded with Hi-C data in YaHS ( Zhou *et al.*, 2023), using the --break option for handling potential misassemblies. The scaffolded assemblies were evaluated using Gfastats ( Formenti *et al.*, 2022), BUSCO ( Manni *et al.*, 2021) and MerquryFK ( Rhie *et al.*, 2020).

The mitochondrial genome was assembled using MitoHiFi ( Uliano-Silva *et al.*, 2023).

### Assembly curation

The assembly was decontaminated using the Assembly Screen for Cobionts and Contaminants ( ASCC) pipeline. TreeVal was used to generate the flat files and maps for use in curation. Manual curation was conducted primarily in PretextView and HiGlass ( Kerpedjiev *et al.*, 2018). Scaffolds were visually inspected and corrected as described by Howe *et al*. (2021). Manual corrections included nine breaks and 13 joins. This reduced the scaffold count by 1.6%. The curation process is described at https://gitlab.com/wtsi-grit/rapid-curation . PretextSnapshot was used to generate a Hi-C contact map of the final assembly.

### Assembly quality assessment

The MerquryFK tool ( Rhie *et al.*, 2020) was run in a Singularity container ( Kurtzer *et al.*, 2017) to evaluate *k*-mer completeness and assembly quality for both haplotypes using the *k*-mer database ( *k* = 31) computed prior to genome assembly. The analysis outputs included assembly QV scores and completeness statistics.

The genome was analysed using the BlobToolKit pipeline, a Nextflow implementation of the earlier Snakemake version ( Challis *et al.*, 2020). The pipeline aligns PacBio reads using minimap2 ( Li, 2018) and SAMtools ( Danecek *et al.*, 2021) to generate coverage tracks. It runs BUSCO ( Manni *et al.*, 2021) using lineages identified from the NCBI Taxonomy ( Schoch *et al.*, 2020). For the three domain-level lineages, BUSCO genes are aligned to the UniProt Reference Proteomes database ( Bateman *et al.*, 2023) using DIAMOND blastp ( Buchfink *et al.*, 2021). The genome is divided into chunks based on the density of BUSCO genes from the closest taxonomic lineage, and each chunk is aligned to the UniProt Reference Proteomes database with DIAMOND blastx. Sequences without hits are chunked using seqtk and aligned to the NT database with blastn ( Altschul *et al.*, 1990). The BlobToolKit suite consolidates all outputs into a blobdir for visualisation. The BlobToolKit pipeline was developed using nf-core tooling ( Ewels *et al.*, 2020) and MultiQC ( Ewels *et al.*, 2016), with containerisation through Docker ( Merkel, 2014) and Singularity ( Kurtzer *et al.*, 2017).

## Genome sequence report

### Sequence data

**Figure 2.**
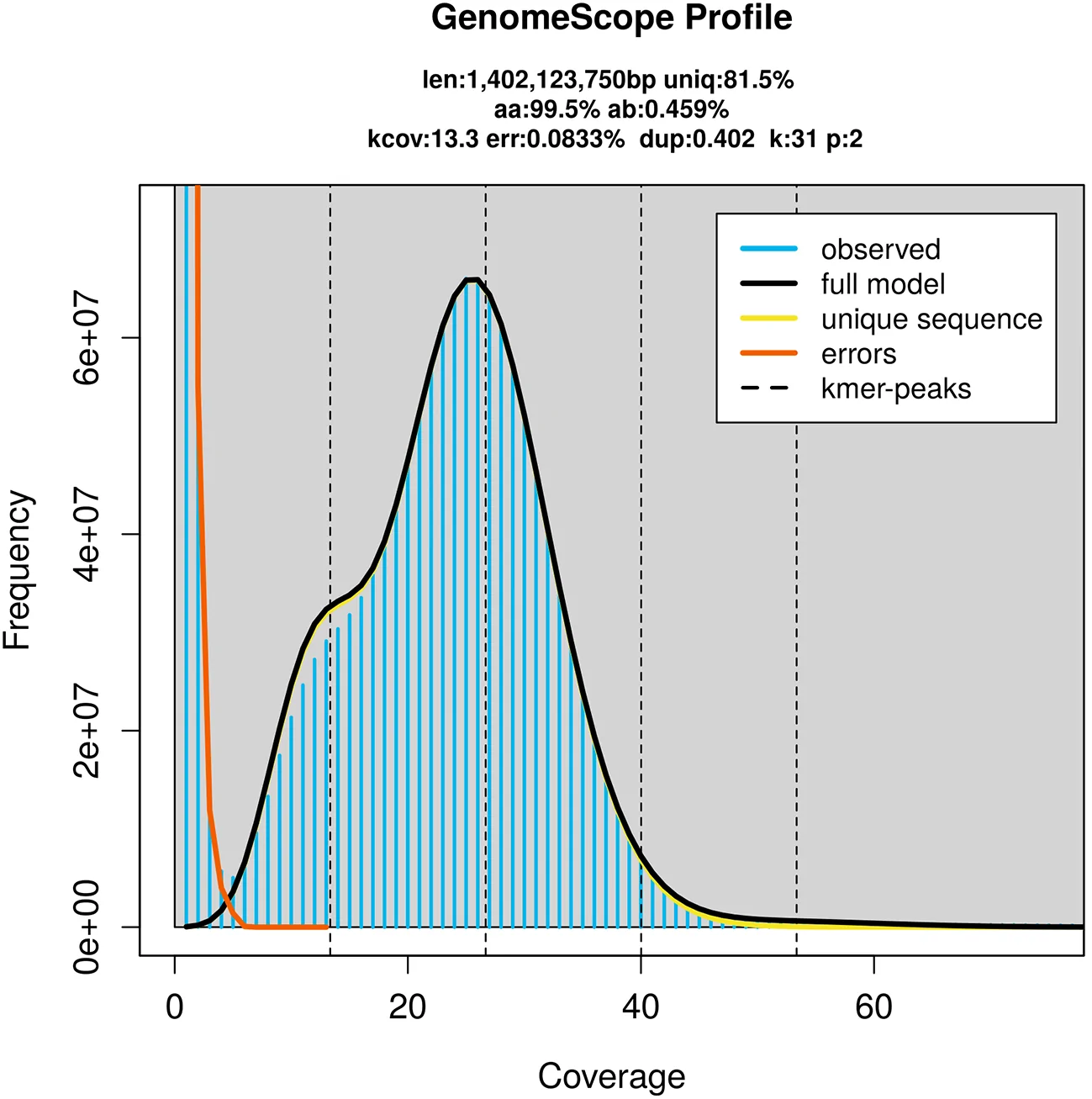
Frequency distribution of *k*-mers generated using GenomeScope2. The plot shows observed and modelled *k*-mer spectra, providing estimates of genome size, heterozygosity, and repeat content based on unassembled sequencing reads.

PacBio sequencing of the *Zootoca vivipara* specimen generated 38.47 Gb (gigabases) from 2.93 million reads, which were used to assemble the genome. GenomeScope2.0 analysis estimated the haploid genome size at 1 403.27 Mb, with a heterozygosity of 0.46% and repeat content of 18.52% ( Figure 2). These estimates guided expectations for the assembly. Based on the estimated genome size, the sequencing data provided approximately 27× coverage. Hi-C sequencing produced 168.53 Gb from 558.05 million reads, which were used to scaffold the assembly. RNA sequencing data were also generated and are available in public sequence repositories. Table 1 summarises the specimen and sequencing details.

**Table 1.** Specimen and sequencing data for *Zootoca vivipara* (BioProject PRJEB65185).

Platform	PacBio HiFi	Hi-C	RNA-seq
**ToLID**	rZooViv2	rZooViv2	rZooViv1
**Specimen ID**	SAN00002413	SAN00002413	SAN00002412
**BioSample (source individual)**	SAMEA111528663	SAMEA111528663	SAMEA111528662
**BioSample (tissue)**	SAMEA111528691	SAMEA111528692	SAMEA111528681
**Tissue**	muscle	muscle	muscle
**Instrument**	Revio	Illumina NovaSeq X	Illumina NovaSeq 6000
**Run accessions**	ERR14708140	ERR11872531; ERR14711434	ERR11872532
**Read count total**	2.93 million reads	558.05 million read pairs	62.10 million read pairs
**Base count total**	38.47 Gb	168.53 Gb	9.37 Gb

### Assembly statistics

The genome was assembled into two haplotypes using Hi-C phasing. Haplotype 1 was curated to chromosome level, while haplotype 2 was assembled to scaffold level. The final assembly has a total length of 1 424.51 Mb in 60 scaffolds, with 108 gaps, and a scaffold N50 of 95.24 Mb ( Table 2).

**Table 2.** Genome assembly statistics for *Zootoca vivipara.*

Genome assembly	Haplotype 1	Haplotype 2
**Assembly name**	rZooViv2.hap1.1	rZooViv2.hap2.1
**Assembly accession**	GCA_965207765.1	GCA_965208345.1
**Assembly level**	chromosome	chromosome
**Span (Mb)**	1 424.51	1 423.70
**Number of chromosomes**	18	18
**Number of contigs**	168	169
**Contig N50**	35.9 Mb	25.04 Mb
**Number of scaffolds**	60	67
**Scaffold N50**	95.24 Mb	95.16 Mb
**Longest scaffold length (Mb)**	134.67	135.08
**Sex chromosomes**	Z _1_ and Z _2_	Z _1_ and Z _2_
**Organelles**	Mitochondrial genome: 17.09 kb	-

**Figure 3.**
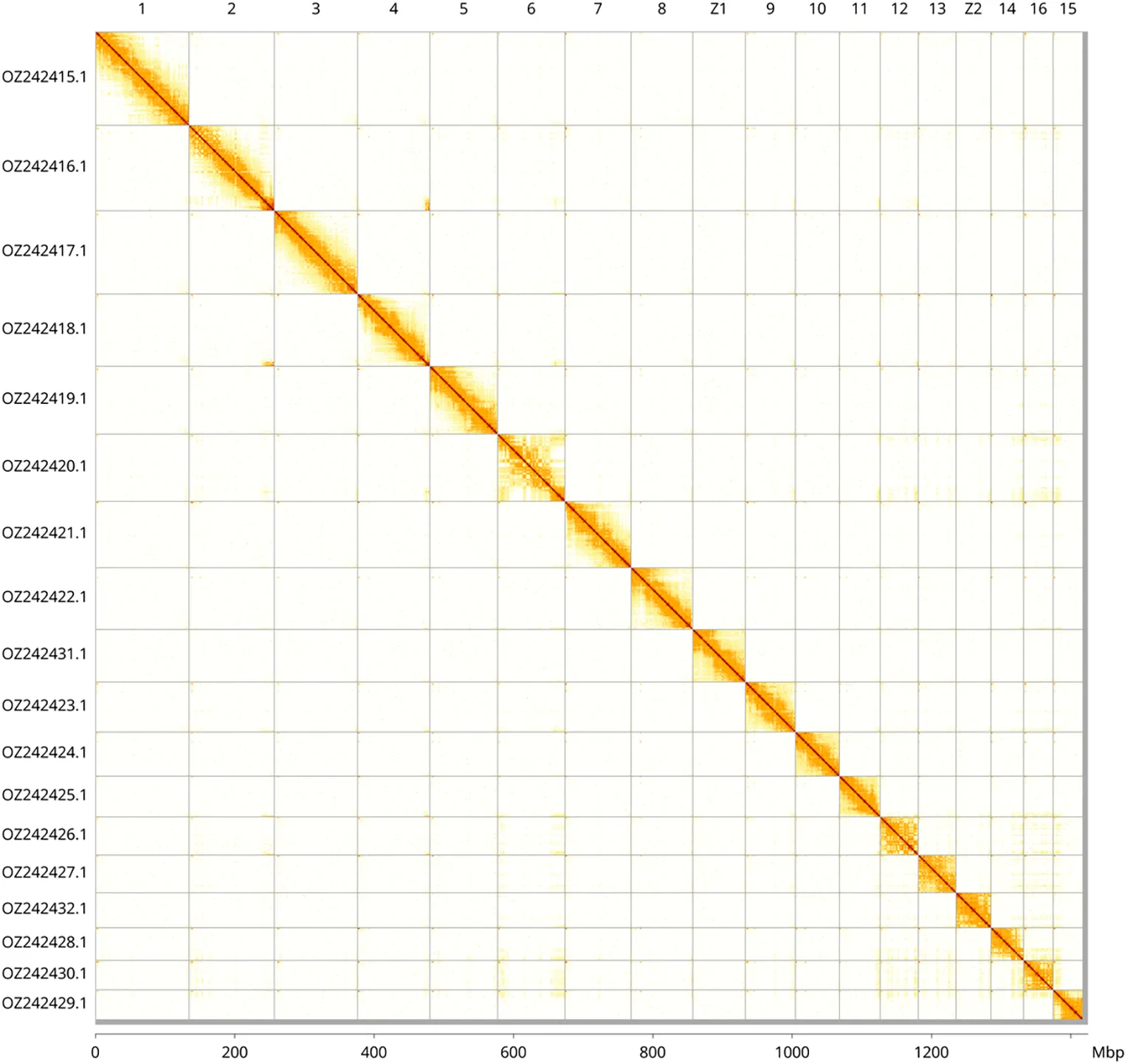
Hi-C contact map of the *Zootoca vivipara* genome assembly. Assembled chromosomes are shown in order of size and labelled along the axes, with a megabase scale shown below. The plot was generated using PretextSnapshot.

Most of the haplotype 1 assembly sequence (99.49%) was assigned to 18 chromosomal-level scaffolds, representing 16 autosomes and the Z _1_ and Z _2_ sex chromosomes. These chromosome-level scaffolds, confirmed by Hi-C data, are named according to synteny ( Figure 3; Table 3).

**Table 3.** Chromosomal pseudomolecules in both haplotypes of the genome assembly of *Zootoca vivipara*, rZooViv2.

Haplotype 1				Haplotype 2			
INSDC accession	Name	Length (Mb)	GC%	INSDC accession	Name	Length (Mb)	GC%
OZ242415.1	1	134.67	43.50	OZ242397.1	1	135.08	43.50
OZ242416.1	2	122.44	44	OZ242398.1	2	122.32	44
OZ242417.1	3	119.49	43	OZ242399.1	3	119.69	43
OZ242418.1	4	103.39	43	OZ242400.1	4	102.39	43
OZ242419.1	5	97.35	43.50	OZ242401.1	5	97.31	43.50
OZ242420.1	6	96.67	46	OZ242402.1	6	96.32	46
OZ242421.1	7	95.24	43.50	OZ242403.1	7	95.16	43.50
OZ242422.1	8	88.13	43	OZ242404.1	8	88.29	43
OZ242423.1	9	71.77	43.50	OZ242405.1	9	71.86	43.50
OZ242424.1	10	63.32	43.50	OZ242406.1	10	62.91	43.50
OZ242425.1	11	58.54	43.50	OZ242407.1	11	59.34	43.50
OZ242426.1	12	54.58	45.50	OZ242408.1	12	54.90	45.50
OZ242427.1	13	54.06	45.50	OZ242409.1	13	53.89	45.50
OZ242428.1	14	46.75	45.50	OZ242410.1	14	46.51	45.50
OZ242429.1	15	42.34	44.50	OZ242411.1	15	42.32	44.50
OZ242430.1	16	42.66	47	OZ242412.1	16	42.53	46.50
OZ242431.1	Z1	75.68	43.50	OZ242413.1	Z1	74.88	43
OZ242432.1	Z2	50.12	44.50	OZ242414.1	Z2	49.73	44.50

The mitochondrial genome was also assembled (length 17.09 kb, OZ242433.1). This sequence is included as a contig in the multifasta file of the genome submission and as a standalone record.

### Assembly quality metrics

**Figure 4.**
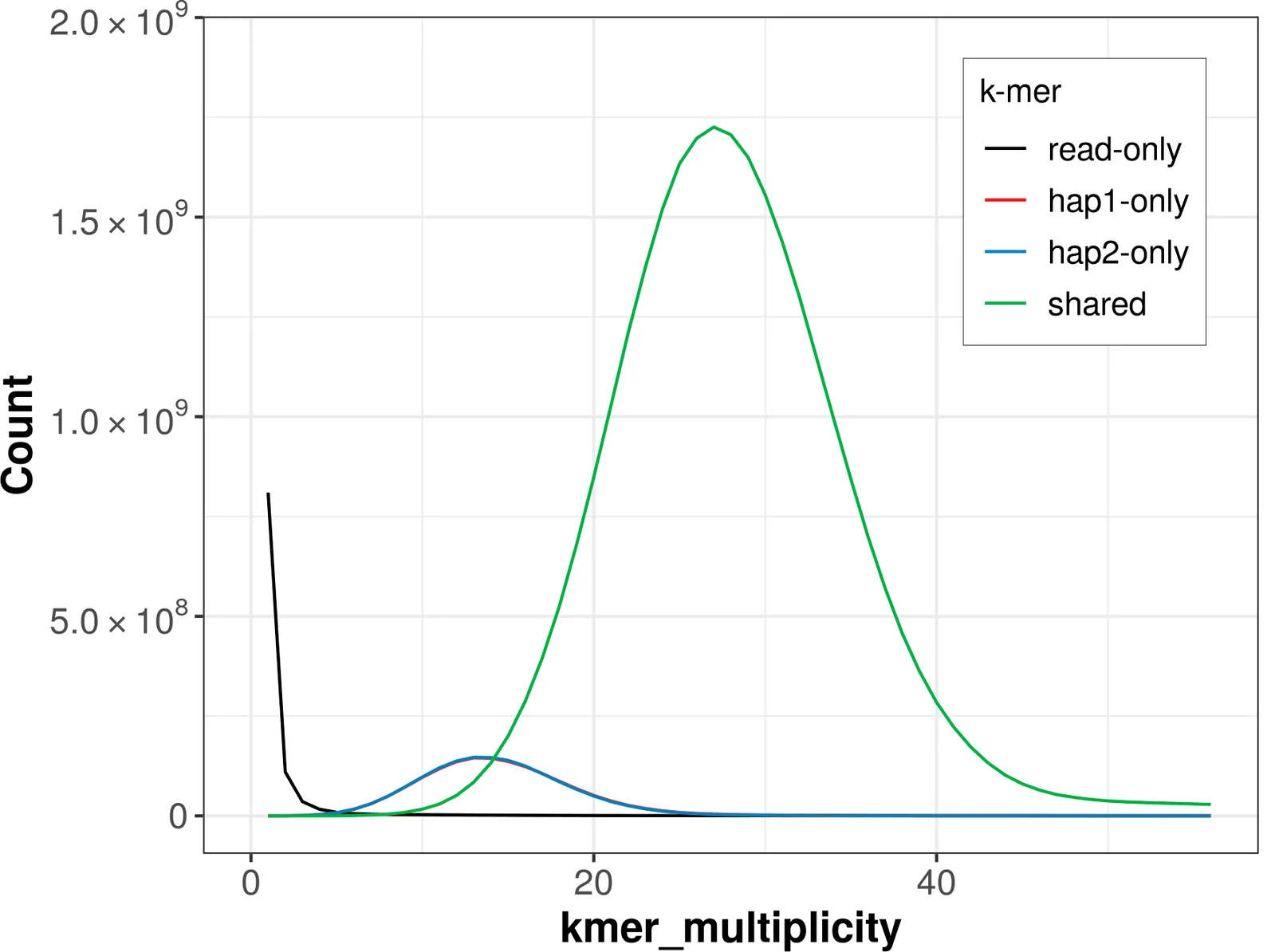
Evaluation of *k*-mer completeness using MerquryFK. This plot illustrates the recovery of *k*-mers from the original read data in the final assemblies. The horizontal axis represents *k*-mer multiplicity, and the vertical axis shows the number of *k*-mers. The black curve represents *k*-mers that appear in the reads but are not assembled. The green curve corresponds to *k*-mers shared by both haplotypes, and the red and blue curves show *k*-mers found only in one of the haplotypes.

For haplotype 1, the estimated QV is 58.2, and for haplotype 2, 58.2. When the two haplotypes are combined, the assembly achieves an estimated QV of 58.2. The *k*-mer completeness is 90.83% for haplotype 1, 90.85% for haplotype 2, and 99.68% for the combined haplotypes ( Figure 4).

**Figure 5.**
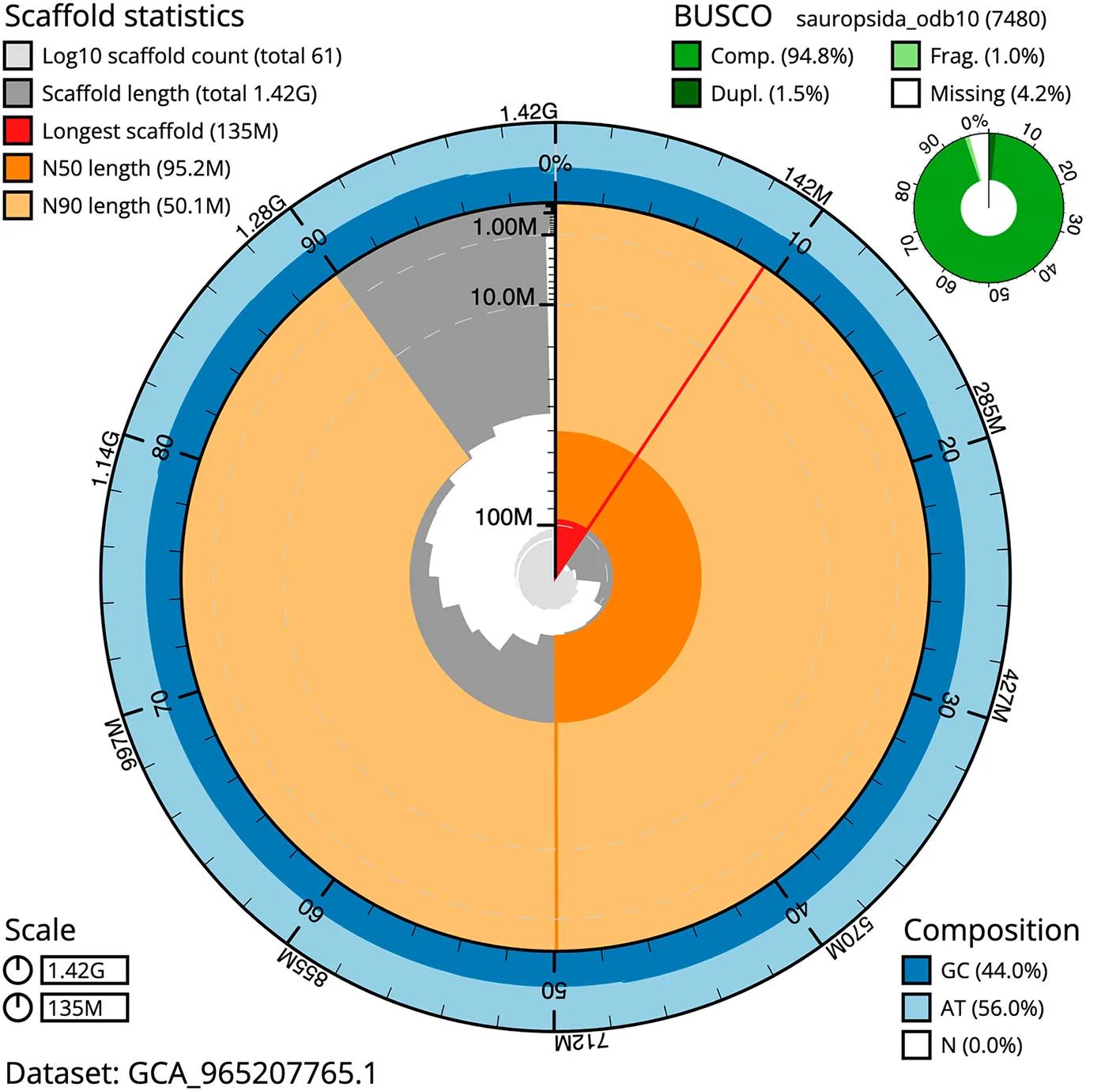
Assembly metrics for rZooViv2.hap1.1. The BlobToolKit snail plot provides an overview of assembly metrics and BUSCO gene completeness. The circumference represents the length of the whole genome sequence, and the main plot is divided into 1 000 bins around the circumference. The outermost blue tracks display the distribution of GC, AT, and N percentages across the bins. Scaffolds are arranged clockwise from longest to shortest and are depicted in dark grey. The longest scaffold is indicated by the red arc, and the deeper orange and pale orange arcs represent the N50 and N90 lengths. A light grey spiral at the centre shows the cumulative scaffold count on a logarithmic scale. A summary of complete, fragmented, duplicated, and missing BUSCO genes in the set is presented at the top right. An interactive version of this figure can be accessed on the BlobToolKit viewer.

**Figure 6.**
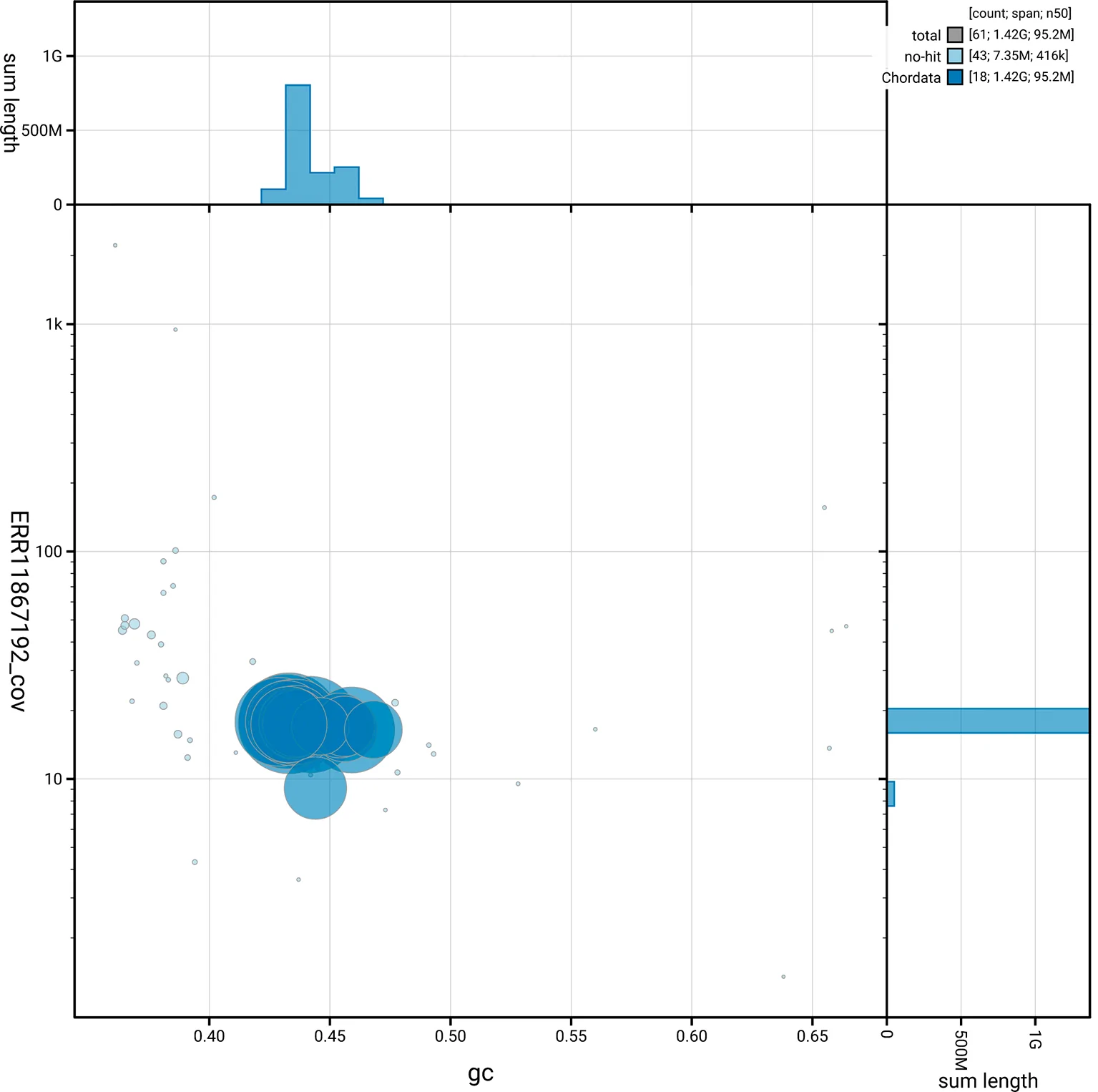
BlobToolKit blob plot for rZooViv2.hap1.1. The plot shows base coverage (vertical axis) and GC content (horizontal axis). The circles represent scaffolds, with the size proportional to scaffold length and the colour representing phylum membership. The histograms along the axes display the total length of sequences distributed across different levels of coverage and GC content. An interactive version of this figure is available on the BlobToolKit viewer.

BUSCO analysis using the sauropsida_odb10 reference set ( *n* = 7 480) identified 94.8% of the expected gene set (single = 93.3%, duplicated = 1.5%) in haplotype 1. For haplotype 2, BUSCO v.5.7.1 analysis identified 94.8% of the expected gene set (single = 93.2%, duplicated = 1.7%). The snail plot in Figure 5 summarises the scaffold length distribution and other assembly statistics for haplotype 1. The blob plot in Figure 6 shows the distribution of scaffolds by GC proportion and coverage for haplotype 1.

Table 4 lists the assembly metric benchmarks adapted from Rhie *et al.* (2021) and the Earth BioGenome Project Report on Assembly Standards. The EBP metric, calculated for the haplotype 1, is **7.C.Q58**, meeting the recommended 6.C.Q40 reference standard.

**Table 4.** Earth Biogenome Project summary metrics for the *Zootoca vivipara* assembly.

Measure	Value	Benchmark
EBP summary (haplotype 1)	7.C.Q58	6.C.Q40
Contig N50 length	35.90 Mb	≥ 1 Mb
Scaffold N50 length	95.24 Mb	= chromosome N50
Consensus quality (QV)	Haplotype 1: 58.2; haplotype 2: 58.2; combined: 58.2	≥ 40
*k*-mer completeness	Haplotype 1: 90.83%; Haplotype 2: 90.85%; combined: 99.68%	≥ 95%
BUSCO	Haplotype 1: C:94.8% [S:93.3%, D:1.5%], F:1.0%, M:4.2%, n:7 480; Haplotype 2: C:94.8% [S:93.2%, D:1.7%], F:0.9%, M:4.2%, n:7 480	S > 90%; D < 5%
Percentage of assembly assigned to chromosomes	99.49%	≥ 90%

*Notes:* The EBP summary uses log10(Contig N50); chromosome-level (C) or log10(Scaffold N50); Q (Merqury QV). BUSCO: C = complete; S = single-copy; D = duplicated; F = fragmented; M = missing; n = orthologues.

**Table 5.** Software versions and sources used for *Zootoca vivipara.*

Software	Version	Source
BLAST	2.14.0	ftp://ftp.ncbi.nlm.nih.gov/blast/executables/blast+/
BlobToolKit	4.4.5	https://github.com/blobtoolkit/blobtoolkit
BUSCO	5.7.1	https://gitlab.com/ezlab/busco
bwa-mem2	2.2.1	https://github.com/bwa-mem2/bwa-mem2
DIAMOND	2.1.8	https://github.com/bbuchfink/diamond
fasta_windows	0.2.4	https://github.com/tolkit/fasta_windows
FastK	1.1	https://github.com/thegenemyers/FASTK
GenomeScope2.0	2.0.1	https://github.com/tbenavi1/genomescope2.0
Gfastats	1.3.6	https://github.com/vgl-hub/gfastats
Hifiasm	0.19.8-r603	https://github.com/chhylp123/hifiasm
HiGlass	1.13.4	https://github.com/higlass/higlass
MerquryFK	1.1.2	https://github.com/thegenemyers/MERQURY.FK
Minimap2	2.28-r1209	https://github.com/lh3/minimap2
MitoHiFi	3	https://github.com/marcelauliano/MitoHiFi
MultiQC	1.14; 1.17 and 1.18	https://github.com/MultiQC/MultiQC
Nextflow	24.10.4	https://github.com/nextflow-io/nextflow
PretextSnapshot	0.0.5	https://github.com/sanger-tol/PretextSnapshot
PretextView	1.0.3	https://github.com/sanger-tol/PretextView
samtools	1.21	https://github.com/samtools/samtools
sanger-tol/ascc	0.1.0	https://github.com/sanger-tol/ascc
sanger-tol/blobtoolkit	v0.7.1	https://github.com/sanger-tol/blobtoolkit
sanger-tol/curationpretext	1.4.2	https://github.com/sanger-tol/curationpretext
Seqtk	1.3	https://github.com/lh3/seqtk
Singularity	3.9.0	https://github.com/sylabs/singularity
TreeVal	1.4.0	https://github.com/sanger-tol/treeval
YaHS	1.2.2	https://github.com/c-zhou/yahs

## Author information

Contributors are listed at the following links:

• Members of the Wellcome Sanger Institute Tree of Life Management, Samples and Laboratory team
• Members of Wellcome Sanger Institute Scientific Operations – Sequencing Operations
• Members of the Wellcome Sanger Institute Tree of Life Core Informatics team
• Members of the Tree of Life Core Informatics collective
• Members of the Darwin Tree of Life Consortium

## Wellcome Sanger Institute – Legal and Governance

The materials that have contributed to this genome note have been supplied by a Darwin Tree of Life Partner. The submission of materials by a Darwin Tree of Life Partner is subject to the **‘Darwin Tree of Life Project Sampling Code of Practice’**, which can be found in full on the Darwin Tree of Life website. By agreeing with and signing up to the Sampling Code of Practice, the Darwin Tree of Life Partner agrees they will meet the legal and ethical requirements and standards set out within this document in respect of all samples acquired for, and supplied to, the Darwin Tree of Life Project. Further, the Wellcome Sanger Institute employs a process whereby due diligence is carried out proportionate to the nature of the materials themselves, and the circumstances under which they have been/are to be collected and provided for use. The purpose of this is to address and mitigate any potential legal and/or ethical implications of receipt and use of the materials as part of the research project, and to ensure that in doing so we align with best practice wherever possible. The overarching areas of consideration are:

• Ethical review of provenance and sourcing of the material
• Legality of collection, transfer and use (national and international)

Each transfer of samples is further undertaken according to a Research Collaboration Agreement or Material Transfer Agreement entered into by the Darwin Tree of Life Partner, Genome Research Limited (operating as the Wellcome Sanger Institute), and in some circumstances, other Darwin Tree of Life collaborators.

## Data Availability

European Nucleotide Archive: Zootoca vivipara (common lizard). Accession number PRJEB65185; https://identifiers.org/ena.embl/PRJEB65185. The genome sequence is released openly for reuse. The *Zootoca vivipara* genome sequencing initiative is part of the Darwin Tree of Life Project (PRJEB40665), the Sanger Institute Tree of Life Programme (PRJEB43745) and the Vertebrate Genomes Project (PRJNA489243). All raw sequence data and the assembly have been deposited in INSDC databases. The genome will be annotated using available RNA-Seq data and presented through the Ensembl pipeline at the European Bioinformatics Institute. Raw data and assembly accession identifiers are reported in Tables 1 and 2. Production code used in genome assembly at the WSI Tree of Life is available at https://github.com/sanger-tol . Table 5 lists software versions used in this study.
