# Supplementary material for: The genome sequence of the Eurasian common lizard, Zootoca vivipara (von Jacquin, 1787) (Squamata: Lacertidae)
Source: Wellcome Open Res. 2026 Jun 11;11:352. [Version 1] doi: 10.12688/wellcomeopenres.26692.1 (PMC13560796; doi:10.12688/wellcomeopenres.26692.1)
Supplement: Peer review history

# Reviewer report: Reviewer response for version 1

**DOI:** 10.21956/wellcomeopenres.29393.r165077

**Published:** 2026-08-25

**Authors:** Arthur Georges

**Affiliations:** 1 Institute for Applied Ecology, University of Canberra, Canberra, Australian Capital Territory, Australia

This is an interesting addition to the genome assemblies for reptiles because it is for a species that has recently evolved viviparity. As such it will provide an excellent model for examining the evolution of regulatory processes involved in the transition from egglaying to livebearing. This is a topic subject of considerable interest.

 The assembly is based on HiFi and HiC without the benefit of ONT UL reads. It appears to have been thoroughly analysed in accordance with the EBP guidelines. The HiC contact map is very clean with the exception of scaffold 20 (ribosomal DNA repeats?). I have no issue with the assembly.

 A few minor technicalities.

 Possible mis-citation? The authors should check Recknagel et al. 2021b ( *J Evol Biol* 34:1167–1176, jeb.13886). This is a broad squamate-wide paleoclimate analysis; it never makes a species-specific superlative claim about *Zootoca*. The near-identical sentence actually appears in Recknagel et al. 2018 ( *Mol Phylogenet Evol*): *"As one of the youngest transitions from oviparity (egg-laying) to viviparity (live-bearing) known in vertebrates (Pyron and Burbrink, 2014; Surget-Groba et al., 2006), common lizards are an emerging model system…"*  -- note in passing that the source says, "known in vertebrates" not "known in any amniote." 

 Are the two morphologically similar? Roitberg 2025 says that viviparous populations exhibited a larger female size than oviparous populations with higher sexual size dimorphism. Maybe qualify the morphologically similar statement?

Are sufficient details of methods and materials provided to allow replication by others?

Yes

Is the rationale for creating the dataset(s) clearly described?

Yes

Are the datasets clearly presented in a useable and accessible format?

Yes

Are the protocols appropriate and is the work technically sound?

Yes

Reviewer Expertise:

Genetics and genomics of sex determination in reptiles.

I confirm that I have read this submission and believe that I have an appropriate level of expertise to confirm that it is of an acceptable scientific standard.

# Reviewer report: Reviewer response for version 1

**DOI:** 10.21956/wellcomeopenres.29393.r158381

**Published:** 2026-06-29

**Authors:** Raul Araya Donoso

**Affiliations:** 1 Yale University, New Haven, USA; 2 Centro de Investigación de Estudios Avanzados del Maule, Universidad Catolica del Maule (Ringgold ID: 28048), Talca, Maule Region, Chile

The authors did a great job describing their study system and setting up its biological relevance. It was really nice to see a well-structured and easy-to-follow introduction.

 I think it should be mentioned in the introduction that a previous reference genome assembly exists for this species (Yurchenko et al., 2020, Genome Biology and Evolution), and that the current study presents a novel, higher-quality phased assembly. It may also be worth discussing how this version improves the previous one.

 Assembly statistics: “These chromosome-level scaffolds, confirmed by Hi-C data, are named according to synteny” – synteny with?

Are sufficient details of methods and materials provided to allow replication by others?

Partly

Is the rationale for creating the dataset(s) clearly described?

Yes

Are the datasets clearly presented in a useable and accessible format?

Yes

Are the protocols appropriate and is the work technically sound?

Yes

Reviewer Expertise:

Evolutionary Genomics

I confirm that I have read this submission and believe that I have an appropriate level of expertise to confirm that it is of an acceptable scientific standard.
